# Bridging the human resource gap in surgical and anesthesia care in low-resource countries: a review of the task sharing literature

**DOI:** 10.1186/s12960-017-0248-6

**Published:** 2017-11-07

**Authors:** Tigistu Ashengo, Alena Skeels, Elizabeth J. H. Hurwitz, Eric Thuo, Harshad Sanghvi

**Affiliations:** 1 Jhpiego, 1615 Thames Street, Baltimore, MD 21231 USA; 2 Johns Hopkins School of Public Health, 615 N. Wolfe, Baltimore, MD 21205 USA; 3 St. Paul Medical College, Gulele Sub-City, Addis Ababa, Ethiopia

**Keywords:** Task sharing, Surgery, Anesthesia

## Abstract

Task sharing, the involvement of non-specialists (non-physician clinicians or non-specialist physicians) in performing tasks originally reserved for surgeons and anesthesiologists, can be a potent strategy in bridging the vast human resource gap in surgery and anesthesia and bringing needed surgical care to the district level especially in low-resource countries. Although a common practice, the idea of assigning advanced tasks to less-specialized workers remains a subject of controversy. In order to optimize its benefits, it is helpful to understand the current task sharing landscape, its challenges, and its promise.

We performed a literature review of PubMed, EMBASE, and gray literature sources for articles published between January 1, 1996, and August 1, 2016, written in English, with a focus on task sharing in surgery or anesthesia in low-resource countries. Gray literature sources are defined as articles produced outside of a peer-reviewed journal. We sought data on the nature and forms of task sharing (non-specialist cadres involved, surgical/anesthesia procedures shared, approaches to training and supervision, and regulatory and other efforts to create a supportive environment), impact of task sharing on delivery of surgical services (effect on access, acceptability, cost, safety, and quality), and challenges to successful implementation.

We identified 40 published articles describing task sharing in surgery and anesthesia in 39 low-resource countries in Africa and Asia. All countries had a cadre of non-specialists providing anesthesia services, while 13 had cadres providing surgical services. Six countries had non-specialists performing major procedures, including Cesarean sections and open abdominal surgeries. While most cadres were recognized by their governments as service providers, very few had scopes of practice that included task sharing of surgery or anesthesia.

Key challenges to effective task sharing include specialists’ concern about safety, weak training strategies, poor or unclear career pathways, regulatory constraints, and service underutilization. Concrete recommendations are offered.

## Background

The Lancet Commission on Global Surgery estimates that five billion people lack access to safe, affordable surgical care, with low-resource countries paying the highest cost in lives lost [1]. This disparity reflects a convergence of health systems and workforce challenges; among these challenges is the shortage of workforce. Surgical interventions are often considered complex procedures to be undertaken only by highly trained specialists, but these cadres are rare in many low-resource settings.

For instance, Africa and Southeast Asia are home to only 12% of the global surgical specialists (surgeons, anesthesiologists, and obstetricians), despite harboring a third of the world’s population. Surgical specialist density in these countries is only 0.7 per 100 000; a minimum density of 20 per 100 000 is considered necessary to tackle the burden of surgical disease [2]. In the gap, surgically treatable conditions can become fatal. A rapid scale-up of qualified surgical and anesthesia providers is critical.

A key approach to tackle the deficit has been using non-specialists to perform procedures traditionally in the domain of specialists. In fact, non-specialists are routinely trained in basic surgical procedures, such as Cesarean section. Task shifting (the allocation of surgical responsibility to non-specialist cadres) and task sharing (the sharing of joint surgical responsibility between specialists and non-specialists, under the oversight of specialists) [1] were established as an interim mechanism to plug the specialist gap but have since evolved to become the mainstay of surgical care delivery in many countries.

Trained non-specialists in Mozambique and Malawi, for example, perform over 90% of major surgical procedures at the district level with similar outcomes to specialists and with substantially higher job retention rates [3, 4]. While these examples argue in favor of scaled task sharing to increase district-level access to safe surgery, the provision of surgical services by non-specialists must also demonstrate cost-effectiveness and acceptability to patients, policy makers, and other health providers.

Assigning markedly complex tasks to less-specialized health workers continues to face significant challenges. The controversy is driven by questions of surgical quality and safety and fears of creating a two-tiered system of care, with “inferior” and “superior” tracks. But other questions also pose challenges: Which procedures should be task shared, and which should remain the proprietary domain of surgeons? How does adding scope and complexity to the non-specialist’s role affect his or her professional standing, and with what impact on employee retention and the appeal of entry to the cadre? (And, conversely, how does an improved status for non-specialists affect specialists and the appeal of the surgical specialties?) How can ministries of health ensure that non-specialists have the right knowledge, skills, and supervision in the context of rapid scaling of the workforce? How is “task creep,” the incremental expansion of a cadre’s scope of practice, prevented? [1] And, underlying all these questions: should task sharing be considered a short- or long-term solution to an intractable workforce challenge? We hope technology disruption (new and novel technology), as mentioned in the *Harvard Business Review* [5], can translate to workforce disruption in low-resource settings, including the acknowledgement that industry leaders very well may not be the ones to develop the simplest and most accessible idea.

We sought to address the gap in knowledge around the barriers to task sharing by providing a structured and comprehensive analysis and proposing solutions.

## Methods

A systematic literature review was conducted on PubMed and EMBASE, and hand searches were completed from bibliographies for articles published in English between January 1, 1996, and August 1, 2016 (20 years). Gray literature sources were consulted as needed to fill in understanding about program models referenced in the peer-reviewed literature. The search applied a combination of key words: task sharing or task shifting, plus non-physicians (clinical officer and associate clinicians), surgery, anesthesia, or obstetrics.

**Fig. 1:**
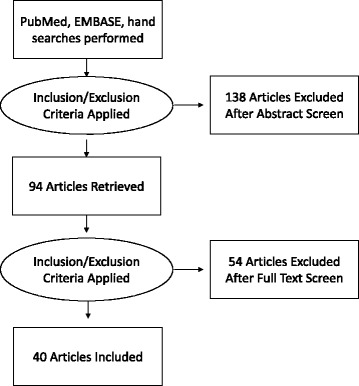
Flow chart: literature selection

Our search produced 232 unique articles. Articles were excluded upon review of the abstract (*n* = 138) or full text (*n* = 54) if they did not cover the nature and forms of task sharing in surgery or anesthesia. Included articles might discuss the involved cadres; the effect of task sharing on surgical access, cost, cost-effectiveness, and patient outcomes; regulation of non-specialists; enablers and barriers to task sharing; and proposed solutions. Observational and quasi-experimental studies and reviews were included. References from selected articles were hand-searched to identify other relevant literature (Fig. 1). Articles published before 1996 and those covering task sharing in non-surgical disciplines were excluded. Data was analyzed by country and then by cadre name, preservice education level, specialized training, surgical procedure, regulation system, and geographic location (Table 1). Procedures performed by non-specialists are analyzed in Table 2.

**Table 1:** Task sharing country profiles: non-physician clinicians involved in task sharing or surgical and anesthesia procedures

Country	Author	Cadre name	Preservice training	Specialized training	Surgical roles/procedures	Regulation system	Geographic location
Eastern Africa							
Kenya	Kakande et al. [6]	Clinical officer	3-year diploma in clinical medicine + 1-year internship	1–1.5-year higher diploma course	Ear, nose, and throat; ophthalmology, anesthesia, orthopedics, minor surgery (e.g., circumcision)	Training and practice regulated by the clinical officers council, act of parliament in place to define roles	Mainly rural
Tanzania	Pereira et al. Beard et al.; McCord et al. [3, 12]	Assistant medical officer	3-year training in clinical medicine + 1-year internship	2-year training for advanced diploma	Emergency obstetric surgery, anesthesia, hernia, abdominal surgery including laparotomy, hydrocelectomy, prostatectomy	Diploma award, accreditation and registration by Tanganyika Medical Council	Urban, rural
Sudan	Kakande et al. [6]	Clinical officer	3-year clinical training		Minor surgery, emergency obstetric surgery, anesthesia		
Uganda	Paul et al.; Galukande et al. [17, 19]	Clinical officer	3-year diploma in clinical medicine + 1-year internship	1–1.5-year higher diploma course	Orthopedics, anesthesia		
Ethiopia	Gessessew et al. [35]	Integrated emergency surgical officer	3–4-year training in clinical medicine, public health, healthcare management	6–9-month training in emergency obstetric care	Emergency obstetric surgery, laparotomy, uterine evacuation, hysterectomy, ophthalmology, anesthesia		
Burundi	McAuliffe et al. [10]	Nurse anesthetist		2-year training program			
Djibouti	McAuliffe et al. [10]	Nurse anesthetist [7]					
Malawi	Wilhelm et al.; Mkandawire et al.; Tyson et al. [4, 25, 30]	Clinical officer	2-year training and 4-year clinical experience as medical assistant	18-month formal training in surgery	Orthopedic, ophthalmology, emergency obstetric surgery, laparotomy	Registration by the Malawi Medical Council,	Urban, rural
Mozambique	Kruk et al.; Cumbi et al. [18, 22]	Surgical technicians Anesthesia technicians	2-year training in clinical medicine as an assistant medical officer + 2–3-year clinical experience	2-year surgical training + 1-year internship for a specialized diploma	Emergency obstetric surgery, orthopedic, general surgery including craniotomy, bowel resection, colostomy, anesthesia	Accreditation by Ministry of Health	Mainly rural
Zambia	Kakande et al. [6]	Medical licentiates	3-year training as clinical officer + 2-year clinical experience	3-year training for a bachelor’s degree in surgery	Anesthesia, orthopedics, emergency obstetric care	Accredited by University of Zambia Regulated by Medical Council of Zambia	Mainly rural
Central Africa							
Cameroon	McAuliffe et al. [10]	Nurse anesthetist	3-year training as registered nurse + 2-year pf experience as intensive care unit nurse	2-year anesthesia training program			
Chad, Angola	McAuliffe et al. [10]	Nurse anesthetist					
West Africa							
Guinea-Bissau	Mullan et al. [8]	Clinical officer	3-year training	N/A	Minor surgery, anesthesia		Mainly rural
Ghana	Mullan et al. [8]	Medical assistant	3-year training + 1-year internship		Minor surgery, emergency obstetric surgery, anesthesia		Mainly rural
Togo	Mullan et al. [8]	Medical assistant			Minor surgery, ophthalmology		
Senegal	Mullan et al. [8]	Health officer	2-year clinical training + 6-month internship		Minor surgery, anesthesia		
Sierra Leone	Mullan et al. [8]	Community health officer Nurse anesthetist	3-year training in community health sciences to earn a higher national diploma in community health sciences	Trainees receive 6 months of surgical training at a central hospital then 6-month rotation to a partner hospital	Cesarean section, laparotomy, hernia repair, hydrocelectomy, appendectomy, tubal ligation, anesthesia		
Burkina Faso	Hounton et al. [20]	Clinical officer	3-year training in clinical medicine and surgery	None	Minor surgery, Cesarean section, ophthalmology		Rural, urban
Côte d’Ivoire	McAuliffe et al. [10]	Nurse anesthetist		2-year training program			
Gabon	Mullan et al. [8] McAuliffe et al. [10]	Nurse anesthetist	Registered nurse with 2-year clinical experience	2-year training program			
Nigeria, Mali, Benin	McAuliffe et al. [10]	Nurse anesthetist					
Southern Africa							
Botswana, Swaziland, Lesotho, Zimbabwe	McAuliffe et al. [10]	Nurse anesthetist					
Central America							
Haiti	Rosseel et al. [26]	Certified nurse	2-year training as a nurse	15–18-month program offered by Medicines San Frontieres (1998–2003) and Partners in Health (2007–)	General anesthesia, spinal anesthesia		
Asia							
Bhutan	Mavalankar et al. [13]	Nurse anesthetist	2-year training as clinical nurse	14-month training combined in Bhutan and Bangkok	General anesthesia, spinal anesthesia		
Afghanistan	Mavalankar et al. [13]	Nurse anesthetist	2-year training as clinical nurse	1 year of training in anesthesia	General anesthesia		
Nepal	Mavalankar et al. [11]	Anesthesia assistant	6-month training	None	General anesthesia		
Cambodia, Laos	Mavalankar et al. [11] McAuliffe et al. [12]	Nurse anesthetist		2-year training			
Philippines, Indonesia, Vietnam, Myanmar, India	McAuliffe et al. [10] Mavalankar et al. [11]	Nurse anesthetist

**Table 2:** Procedures performed by non-specialists in seven surgical specialties

Surgical specialty area	Obstetrics [4, 13, 20, 36, 37]	General surgery [4, 14, 19]	Orthopedics [25]	Ophthalmology [35]	Ear, nose, throat/otolaryngology [6]	Neuro surgery [4]	Plastic surgery [6]
Procedures performed	• C-section • Repair of ruptured uterus • Hysterectomy • Ectopic pregnancy • Tubal ligation • Dilatation and curettage • Manual vacuum aspiration	• Exploratory laparotomy • Inguinal hernia repair • Hydrocelectomy • Appendectomy • Prostatectomy • Bowel surgery (resection, colostomy) • Circumcision • Incision and drainage of abscess • Surgical repair of traumatic wounds	• Debridement of open fractures • External fixation of fractures • Closed fracture manipulation • Joint dislocation • Acute pyogenic musculoskeletal infections • Limb amputation • Management of club foot	• Cataract surgery • Evisceration of the globe • Management of acute eye emergencies • Management of refractive errors [3]	• Ear/nasal foreign body removal • Tonsillectomy • Excision of nasal soft tissue	• Craniotomy • V-P shunting for hydrocephalus	• Management of burn injuries • Skin grafting

## Results

### Common task sharing arrangements

We found reports of performance of surgical and anesthesia tasks by non-specialists across low-resource settings in Africa (29 countries), Asia (10 countries), and Central America (1 country) (Table 1). The practice of task sharing is not only widespread but has become the leading mode of surgical provision for emergency and essential surgeries in some regions.

A common approach to preparing for task sharing involves the training of preexisting cadres for a specialized role. Training occurs through both formal and informal processes. Formal training occurs through recognized channels, either centrally or through a work-site model. It may be funded by governments, religious groups, or other government-sanctioned donors. Informal training occurs through in-service “on-the-job” learning without official sanction or curriculum. Formal programs were noted to run for a period of 6 months to 2 years, sometimes followed by a supervised internship [6, 7].

Task sharing cadres can be classified into two main groups: those with nursing degrees (usually nurse anesthetists) and those trained from non-nursing pathways. Depending on the country, non-nursing cadres are designated different titles such as clinical officer (Kenya, Uganda, and Malawi), assistant medical officer (Tanzania), health officer (Ethiopia), and surgical technician (Mozambique) [6, 8, 9].

The scope of surgical tasks shared varies by country. Malawi, Mozambique, Tanzania, Zambia, Ethiopia, and Sierra Leone demonstrated the widest scope, including non-specialist involvement in the provision of major emergency obstetric surgery (see Table 1 for details). In Mozambique, non-specialists undertake a broad portfolio of both general (laparotomy, including bowel surgery), obstetric (Cesarean section, repair of uterine rupture, and hysterectomy), and orthopedic procedures. While Kenya allows clinical officers to specialize (becoming “non-specialist specialists” in ear, nose, and throat; orthopedics; and ophthalmology), their roles are restricted to conservative procedures [6]. Mid-level providers (nurse anesthetists and clinical officer-anesthetists) were noted to be the predominant providers of both general and spinal anesthesia at the district level across all countries studied [10, 11].

### Effect of task sharing on quality of care

Comparing the quality of emergency obstetric surgery performed by assistant medical officers versus general practitioners in Tanzania, the investigators found no difference in surgically related maternal mortality, perinatal deaths, or complications such as wound infection, burst abdomen, ruptured uterus, fistula, and ureteral injury. [12] Similarly, a study in Malawi comparing Cesarean section performance by clinical officers versus general practitioners reported comparable rates of maternal death, reoperation rate, wound infection, and wound dehiscence [13]. In a meta-analysis comparing the incidence of complications after male circumcision performed by physicians versus non-physicians, the investigators found no difference in the incidence of the two most common complications: bleeding and wound infection [14].

Two Cochrane reviews failed to reach conclusions as to the comparisons between specialists and non-specialists. Lewis et al., comparing the delivery of anesthesia between the two groups, found the data available to be of low quality, rendering it premature to provide a conclusion [15]. In the second review, studying the difference in post-abortion complications, the authors concluded that despite observational studies pointing to a higher risk of surgical abortion failure by non-specialists, the number of studies available is too small to support a conclusion of superior care by physicians [16].

### Acceptability of task sharing

Evidence of acceptability from the perspective of the non-specialist, other healthcare workers, policy decision-makers, and patients was reviewed. Non-specialists find it favorable to perform an extended scope of duties for two main reasons: [1] an altruistic motive to save lives, especially in settings where they happen to be the most qualified provider [17], and [2] they felt that task sharing had salutary effects on job satisfaction emanating from improved clinical skills and confidence; it also opened doors for promotion to administrative roles and enhanced their ability to make extra income in private practice [17, 18]. Factors associated with a negative attitude among non-specialists included increased workload that is not matched with remuneration, poor career progression, and role overlap with specialists [18].

Task sharing seems to confer a positive payoff to specialists mainly in the form of reduced workload that allows them to concentrate on more complex tasks [17, 19]. On the other hand, studies indicate that some specialists find task sharing to be untenable due to concerns over quality of care, ethical reservations about creating a second tier of care for underserved groups, and a perceived loss of power to the mid-level provider [18]. These challenges are discussed in detail under the section on barriers to task sharing. Although government support of training programs for non-specialist cadres and the widespread practice of task sharing at the facility level may be taken as surrogate indicators of the opinion of policy makers, we did not find any studies evaluating this dimension. There were also no studies evaluating patient experiences with these cadres.

### Cost and cost-effectiveness of task sharing

Four studies explored economic elements of task sharing. Three studies focused on cost-effectiveness, and one evaluated the impact of non-specialists on the cost of surgery. The first study evaluated the cost-effectiveness of Cesarean section deliveries performed by clinical officers, general practitioners, and obstetricians by computing the incremental cost-effectiveness ratios using newborn fatality rates [20]. The study concluded that general practitioners were the most cost-effective cadre for task sharing Cesarean sections in this context. In a second study from Malawi, Grimes et al. demonstrate that the training of non-specialists to provide orthopedic surgery is superior in its cost-effectiveness to other routine public health initiatives such as oral rehydration, antiretroviral programs for HIV, and breast feeding promotion. The authors, however, admit shortcomings in accounting for all costs including set-up, equipment, and direct costs to patients. In a different study based in Malawi, the authors estimate the cost of training an orthopedic clinical officer at 7253 US dollars over 18 months (between 1998 and 2007) compared to 52 000 US dollars required to train an orthopedic specialist over 9 years [21].

Kruk et al. compared the cost of training non-specialists and specialists in Mozambique over a 30-year clinical career based on the performance of three core procedures: Cesarean section, obstetric hysterectomy, and laparotomy for ectopic pregnancy. Modeled on an assumption that the decision to operate and complication rates are similar between the two cadres, the cost of training and deployment of non-specialists was substantially lower at 27% that of specialist costs (71 914.80 US dollars versus 167 057.70 US dollars). The cost per surgery performed by a non-specialist was less than half that of a specialist and remained favorable even after the hypothetical doubling of their salaries. The difference, the authors argue, is largely the amount of time to train specialists who in turn command higher salaries [22].

In the fourth study, Shrime et al. evaluated the effect of three policy initiatives in enhancing access to surgical services in Ethiopia: task sharing, universal public funding, and transport vouchers. While task sharing was noted to accrue substantial health benefits, increased uptake of surgery came at a cost of increasing impoverishment among the poorest quintiles, who pay out of pocket for surgery. Impoverishment was partially ameliorated by combining task sharing with universal public funding [23].

### Barriers to safe and effective task sharing in surgery and anesthesia

#### Physician resistance

From the literature surveyed, specialist resistance to substantial involvement of non-specialists in surgical and anesthesia practice is driven by three main issues: perceived erosion of safety and quality of care, change in power dynamics, and ethical concerns. While specialists acknowledged that non-specialists can be successfully trained to perform surgery to achieve a similar level of operating skill, they felt that non-specialists do not have the requisite clinical knowledge to match operative skills [24]. This, they argued, limits the ability of non-specialists to make informed decisions about when or whether to perform surgery and their capacity to respond adequately to rapidly changing clinical situations. Specialists also perceived the expansion of the non-specialist’s role to perform surgical tasks as a threat to their position as the presumed leader of the clinical team. A qualitative study from Mozambique found that specialists viewed non-specialists as subordinate and perceived their advanced surgical skills as a threat to their power [18]. Further grounds for specialist resistance were based on an ethical viewpoint. Here, specialists argue that creation or expansion of a less-skilled workforce targeted at delivering services to marginalized and largely poorer communities creates a tier of second-rate clinical services as the need to expand coverage outweighs the quality and safety of care [24].

#### Training effectiveness

We found three key barriers to effective training: disruption of training due to loss of funding, deficient assurance of skills mastery, and lack of accreditation systems. Malawi’s flagship program providing training to orthopedic clinical officers was stalled between 1995 and 1998 following the closure of a foreign-funded grant and subsequent departure of the program founder from the country [25]. A similar disruption occurred in Haiti’s training of nurse anesthetists following Médecins Sans Frontières closure of its primary care program [26].

As far as the quality of training is concerned, specialists reported that central hospitals were inappropriate training venues for non-specialists. Due to the crowded and busy nature of these facilities, non-specialist trainees had to compete with specialist trainees for surgical cases and attention from supervisors. Additional, central hospitals offered a poor reflection of the population, environment, and resource constraints of the district facilities to which non-specialists would eventually serve [27].

Although surgical task sharing continues to be ubiquitous, many practicing non-specialists acquire their skill informally through exposure at the work place necessitated by the lack of specialists or through non-accredited training programs. While this may expand their scope of practice at their designated facility, lack of formalized professionalization inhibits quality management: programs are often not accredited, the workforce often uncertified, continuing education often scant, supervision and mentorship often haphazard, and skills untested [1].

#### Poor career progression

Poor career progression was identified as a key driver of low morale and low performance among non-specialists [28]. Studies found that the pay of non-specialists was lower than other clinical cadres and remained so even after non-specialists undertook an expanded and more complex surgical mandate. One study reported that non-specialists at the height of their career earned four times less than a newly qualified medical doctor [18]. In Haiti, low salaries in the public health service led to the exit of nurse anesthetists who took up employment in private hospitals that offered more lucrative terms [26].

Members of other clinical cadres (nurses, medical doctors, lab technicians) acknowledged the significant role played by non-specialists, but non-specialists failed to achieve a distinct professional identity [28]. While this has its roots in unofficial, unaccredited training models, it also appears to arise from poor definition of roles, especially when non-specialists work alongside specialists [29]. For instance, in a qualitative study in Kenya, non-specialists expressed dissatisfaction at being assigned general duties despite their surgical training [27].

A lack of opportunities to advance skills and knowledge contribute to job stagnation. Findings from interviews with non-specialists identify few trainings tailored to meet the need for mid-level skills [19]. Even when training is available, it fails to confer a recognizable advancement in competence due to lack of accreditation. Some authors argue that this low investment in training owes to the fact that task sharing is viewed as a short-term solution to a time-limited physician shortage rather than a sustainable, long-term approach to health systems strengthening [24, 30]. Resulting in part from role confusion and lack of opportunity for continuing education, job descriptions and job titles for non-specialists rarely reflect growth, remaining the same through most of their careers [28].

#### Regulatory constraints

Regulatory issues affect task sharing in two distinct ways. First, most task sharing initiatives arose as a stop-gap response to physician shortage and therefore prospered outside of formal regulatory control [6]. Meanwhile, existing medical regulatory regimes preclude the involvement of other cadres and tightly regulate physician practice [31]. To enable task sharing scale-up, revision of surgical regulation is likely necessary. Even within current norms, unregulated practice of surgery and anesthesia may expose patients to safety risks and non-specialists to legal risks [17].

## Recommendations

### Addressing physician resistance

Ministries or national institutions in charge of formulating health policies can address physician resistance in three main ways. First, ministries should engage nursing, physician, and other health provider groups in the planning and implementation of task sharing activities. This is critical to marshal the required support. Involvement from the initial stages in curriculum design would help to delineate roles and ameliorate inefficiencies arising from role overlap among cadres [1].

Second, to reduce role conflict, ministries should clearly define the scopes of practice of non-specialists [1]. A useful strategy for surgery would be to construct a portfolio of priority procedures and develop a framework that stratifies them by complexity, learning curve, and risk. This can facilitate the identification of procedures that may be safely and effectively performed by non-specialists with appropriate training and mentorship. This was done successfully in the Netherlands [31], and the World Health Organization has published recommendations on optimizing health worker roles in task sharing activities for maternal and child health [32].

Third, ministries should consider the use of time-limited local pilot task sharing initiatives ahead of full adoption, as this provides the opportunity to test and tailor new strategies to local needs and to gradually build support prior to full implementation. In the Netherlands, thoughtful legislation to effect task sharing from physicians to nurses, coupled with nationwide evaluation, provided an option to abandon the initiative if it proved ineffective at the end of a 5-year trial and another option for widespread adoption if the initiative was found to be viable. This also had a palliating effect on physician resistance [33].

### Optimizing training effectiveness

Training programs should target selection of candidates from rural backgrounds or those with substantial rural healthcare experience, as this has been shown to enhance chances of retention in areas of greatest need. In Mozambique, for example, candidates for non-specialist training are selected from the strongest nurses with rural experience and have retention rates as high as 90% [18]. There is, however, insufficient evidence to directly link high retention to the selection process. Training centers and clinical residencies (or substantial phases of training) should be situated in areas where resources and limitations match those in the areas to which the workforce will be deployed to assure that training is attuned to the local needs and circumstances. So as to assure sustainability, training programs should be integrated into national health plans. Training disruption in Malawi and Haiti after donor program closure speaks to the need to secure government support and include training programs into national human resource plans to assure sustained funding at the end of donor grant cycles [25, 26]. A thoughtfully designed and implemented system for supervision and mentorship could both enhance patient safety and foster professional development and collaboration.

### Improving career progression

Advancements in career progression for non-specialists can be achieved by conferring a distinct professional identity and ongoing skills improvement pathway that are matched with progressive remuneration. Health ministries and regulatory bodies should accredit training programs to standardize training. This can enhance professional identity by offering a sense of differentiation among non-specialists. For instance, to improve non-specialist standing, Malawi and Mozambique have upgraded their surgical and anesthesia technician training from diploma to Bachelors’ degree level [34]. Studies from Malawi also report the creation of a shorter pathway for experienced non-specialists to enroll in medical school [30]. There are currently no studies to evaluate the effect of these interventions on career progression. In addition, professional societies, with the support of ministries and regulatory bodies, should design and deliver continuous professional development activities. These are not only vital for the maintenance and acquisition of skill but have also been shown to ameliorate the feeling of professional isolation among rural health workers. Ministries should also review job structures to provide career opportunities similar to those of other healthcare workers. This should include matching remuneration and benefits to recognize an expanded role [1].

### Lifting regulatory constraints

Health ministries should advocate for the review of existing medical practice regulations to expand the scope of non-specialists in line with community needs, potentially including legal protection. Government regulation strategies used in Europe and self-regulation in Australia could be helpful [33].

### Limitations

A chief limitation in this study is that findings are based only on the published literature, which offers scant information on implementation models. We may have missed certain cadres if their nomenclature was unknown to us and task sharing or shifting was not referenced.

## Conclusions

The reasons for task sharing range from workforce shortage to cost reduction, which are long-term issues that warrant a robust policy discussion. Task sharing in surgery and anesthesia can enhance access to safe and cost-effective surgery. Although task sharing has often been initiated as a short-term measure, data from numerous countries suggests that it should be viewed as a long-term complementary strategy to the training of surgeons [4, 29]. There is a need for modeling exercises (including cost and lives lost) to identify the optimal workforce mix over time.

While all evidence points to the safety and cost-effectiveness of task sharing, robust studies are needed, especially of what interventions in training, mentorship, oversight, and policy would result in the safe and cost-effective expansion of surgical access. Additional studies on the perception and aspirations of non-specialists would be useful in helping to define effective career pathways. A focus on the needs of patients will help to align integration and collaboration between different cadres in health care, allowing for a clear differentiation of roles including mentorship, supervision, and career development.

We hope this paper contributes not only a compilation of current practice, but also a structured and comprehensive examination of the barriers and challenges implementers will need to address the global workforce crises in surgery. We hope that our recommendations will aid future implementers and that they will work with researchers to isolate the elements of effective task sharing programs so that these programs may be scaled up to address the needs of the five billion people who lack access to safe, affordable surgical care.
